# World addiction medicine reports: formation of the International Society of Addiction Medicine Global Expert Network (ISAM-GEN) and its global surveys

**DOI:** 10.3389/fpsyt.2024.1230318

**Published:** 2024-03-11

**Authors:** Hamed Ekhtiari, Arash Khojasteh Zonoozi, Parnian Rafei, Fateme Sadat Abolghasemi, Dan Pemstein, Tarek Abdelgawad, Sophia Achab, Hamad Al Ghafri, Mustafa Al’Absi, Michaël Bisch, Aldo Alberto Conti, Atul Ambekar, Shalini Arunogiri, Roshan Bhad, Rabia Bilici, Kathleen Brady, Gregory Bunt, Anja Busse, Jenna L. Butner, Ahmad Danesh, Joseph El-Khoury, Fatima El Omari, Darius Jokūbonis, Cor de Jong, Geert Dom, Mohsen Ebrahimi, Ali Fathi Jouzdani, Marica Ferri, Susanna Galea-Singer, Dario Gigena Parker, Susumu Higuchi, Preethy Kathiresan, Emira Khelifa, Christos Kouimtsidis, Evgeny M. Krupitsky, Jiang Long, Icro Maremmani, Garrett McGovern, Hossein Mohaddes Ardabili, Afarin Rahimi-Movaghar, Solomon Tshimong Rataemane, Arshiya Sangchooli, Goodman Sibeko, Anna Maria Vella, Salvador Benjamin D. Vista, Mehran Zare-Bidoky, Min Zhao, Afzal Javed, Marc N. Potenza, Alexander Mario Baldacchino

**Affiliations:** ^1^ Department of Psychiatry, University of Minnesota, Minneapolis, MN, United States; ^2^ Laureate Institute for Brain Research, Tulsa, OK, United States; ^3^ Iranian National Center for Addiction Studies, Tehran University of Medical Sciences, Tehran, Iran; ^4^ Faculty of Medicine, Mashhad University of Medical Sciences, Mashhad, Iran; ^5^ Trinity College Institute of Neuroscience (TCIN), Trinity College Dublin, Dublin, Ireland; ^6^ Political Science and Public Policy & Challey Institute, North Dakota State University, Fargo, ND, United States; ^7^ Faculty of Medicine, Cairo University, Cairo, Egypt; ^8^ Faculty of Medicine, Sociological and Psychological Research Unit, Department of Psychiatry, University of Geneva, Geneva, Switzerland; ^9^ National Rehabilitation Center, Abu Dhabi, United Arab Emirates; ^10^ Pôle Hospitalo-Universitaire de Psychiatrie d’Adultes et d’Addictologie du Grand Nancy, Centre Psychothérapique de Nancy, Laxou, France; ^11^ Department of Child & Adolescent Psychiatry, Institute of Psychiatry, Psychology and Neuroscience, King’s College London, London, United Kingdom; ^12^ National Drug Dependence Treatment Centre, All India Institute of Medical Sciences, New Delhi, India; ^13^ Monash Addiction Research Centre, Eastern Health Clinical School, Melbourne, VIC, Australia; ^14^ Faculty of Medicine, Department of Psychology, Istanbul Ticaret University, Istanbul, Türkiye; ^15^ Department of Psychiatry and Behavioral Sciences, Medical University of South Carolina, Charleston, SC, United States; ^16^ School of Medicine, New York University, New York City, NY, United States; ^17^ Prevention, Treatment and Rehabilitation Section, United Nations Office on Drugs and Crime (UNODC), Vienna, Austria; ^18^ Department of Internal Medicine, Yale School of Medicine, New Haven, CT, United States; ^19^ Division of Prevention Science, University of California, San Francisco, San Francisco, CA, United States; ^20^ Lebanese Psychiatric Society, Beirut, Lebanon; ^21^ Faculty of Medicine, University Mohammed Vth of Rabat, Rabat, Morocco; ^22^ Republican Center for Addictive Disorders, Lithuanian University of Health Sciences, Kaunas, Lithuania; ^23^ Behavioral Science Institute, Radboud University, Nijmegen, Netherlands; ^24^ Collaborative Antwerp Psychiatric Research Institute (CAPRI), University of Antwerp, Antwerp, Belgium; ^25^ European Monitoring Centre for Drugs and Drug Addiction (EMCDDA), Lisbon, Portugal; ^26^ DigitAS Project, Population and Behavioural Science, School of Medicine, University of St Andrews, St Andrews, United Kingdom; ^27^ National Health Service (NHS) Fife Addiction Services, Cameron Hospital, Windygates, United Kingdom; ^28^ School of Public Health, National University of Cordoba, Cordoba, Argentina; ^29^ National Hospital Organization Kurihama Medical and Addiction Center, Yokosuka, Japan; ^30^ Department of Psychiatry, All India Institute of Medical Sciences (AIIMS), New Delhi, India; ^31^ Faculty of Medicine of Tunis, University of Tunis El Manar, Tunis, Tunisia; ^32^ National Office for Addressing Drugs, Athens, Greece; ^33^ Surrey and Borders Partnership, National Health Service (NHS) Foundation Trust, Leatherhead, United Kingdom; ^34^ Department of Medicine, Imperial College London, London, United Kingdom; ^35^ Department of Addictions, Bekhterev National Medical Research Center for Psychiatry and Neurology, St. Petersburg, Russia; ^36^ Faculty of Medicine, University of Malaya, Kuala Lumpur, Malaysia; ^37^ Department of Clinical and Experimental Medicine, Section of Psychiatry, University of Pisa, Pisa, Italy; ^38^ UniCamillus, International Medical University in Rome, Rome, Italy; ^39^ Irish Chapter of International Society of Addiction Medicine (IRE-ISAM), Dublin, Ireland; ^40^ Department of Psychiatry, University of Limpopo, Polokwane, South Africa; ^41^ Melbourne School of Psychological Sciences, University of Melbourne, Melbourne, VIC, Australia; ^42^ Department of Psychiatry and Neuroscience Institute, University of Cape Town, Cape Town, South Africa; ^43^ Foundation for Social Welfare Services (FSWS), Sedqa, Santa Venera, Malta; ^44^ Department of Psychiatry and Behavioral Medicine, College of Medicine and Philippines General Hospital, University of the Philippines Manila, Manila, Philippines; ^45^ Shanghai Mental Health Center, Shanghai Jiaotong University School of Medicine, Shanghai, China; ^46^ Pakistan Psychiatric Research Centre, Fountain House, Lahore, Pakistan; ^47^ Department of Psychiatry and Child Center, Yale School of Medicine, New Haven, CT, United States; ^48^ Connecticut Mental Health Center, New Haven, CT, United States; ^49^ Connecticut Council on Problem Gambling, Wethersfield, CT, United States; ^50^ Wu Tsai Institute and Department of Neuroscience, Yale University, New Haven, CT, United States

**Keywords:** addiction medicine, global, expert, survey, elicitation, network

## Abstract

Addiction medicine is a dynamic field that encompasses clinical practice and research in the context of societal, economic, and cultural factors at the local, national, regional, and global levels. This field has evolved profoundly during the past decades in terms of scopes and activities with the contribution of addiction medicine scientists and professionals globally. The dynamic nature of drug addiction at the global level has resulted in a crucial need for developing an international collaborative network of addiction societies, treatment programs and experts to monitor emerging national, regional, and global concerns. This protocol paper presents methodological details of running longitudinal surveys at national, regional, and global levels through the Global Expert Network of the International Society of Addiction Medicine (ISAM-GEN). The initial formation of the network with a recruitment phase and a round of snowball sampling provided 354 experts from 78 countries across the globe. In addition, 43 national/regional addiction societies/associations are also included in the database. The surveys will be developed by global experts in addiction medicine on treatment services, service coverage, co-occurring disorders, treatment standards and barriers, emerging addictions and/or dynamic changes in treatment needs worldwide. Survey participants in categories of (1) addiction societies/associations, (2) addiction treatment programs, (3) addiction experts/clinicians and (4) related stakeholders will respond to these global longitudinal surveys. The results will be analyzed and cross-examined with available data and peer-reviewed for publication.

## 1 Introduction

### 1.1 Background

Substance use disorders (SUDs) and other addictive behaviors remain among the most crucial global issues affecting approximately 36 million people worldwide. According to the World Drug Report 2021, the number of people using drugs is estimated to rise by 11% by 2030 (1). A central challenge has been that addiction prevention, control and treatment are intertwined with multiple political, socio-economic, and public health considerations, necessitating the engagement of various stakeholders for effective policies and interventions. Addressing drug manufacturing, trade and use as global phenomena requires harmonized national and international action plans and decisions informed by both the available evidence and the opinions of diverse experts. This was highlighted during the COVID-19 pandemic, with new and intensified challenges and a scarcity of evidence to guide timely decision-making (2, 3).

Despite improvements in practices of organizations worldwide, significant differences remain in the quality and quantity of the data available on many substance-related issues across nations, and systemic information and practice gaps remain to be addressed. These discrepancies hamper the development of thorough national and international reports which reflect the unique complexities and challenges of addiction medicine and cover diverse themes such as substance use profiles and the feasibility, accessibility, availability and affordability of interventions, especially substance-related treatments. As one recent example, the International Society for Addiction Medicine (ISAM) rapidly formed a working group in the early days of the COVID-19 pandemic and conducted a global survey of issues related to addictive disorders, and 189 experts in addiction medicine from 72 countries were surveyed after the development of the study protocol (1–3). The survey demonstrated the impact of the pandemic on SUD treatment and harm reduction services. This highlighted several issues and complications affecting people with SUDs (3) and captured important changes in the supply and demand of licit and illicit substances (1).

As mentioned earlier, there have been multiple efforts by international, regional, and national organizations such as the World Health Organization (WHO), United Nations Office on Drugs and Crime (UNODC), European Monitoring Centre for Drugs and Drug Addiction (EMCDDA), Global Drug Survey (GDS), and Substance Abuse and Mental Health Services Administration (SAMHSA) within the U.S. Department of Health and Human Services (HHS) to design and implement surveys to gather data regarding different aspects of SUDs from different reference points, from governmental contact points for the WHO, UNODC, and EMCDDA to local treatment facilities by SAMHSA and drug-using populations by GDS (4–9). Excluding the U.S. National Survey of Substance Abuse Treatment Services (N-SSATS) conducted by SAMHSA which is mainly focused on substance-related treatment facilities in the US, most other regional and international reports do not collect data from clinicians, treatment programs, and their organizations. As an example, the UNODC’s Annual Report Questionnaire (ARQ) collects data on the extent and pattern of drug use, demand, crop cultivation, manufacturing, and trafficking with a limited number of general questions on substance use treatment profiles obtained from the governmental contact points (5, 6). The EMCDDA’s datasets include sections regarding treatment demands in European countries that describe general demographic features related to receiving treatment such as gender, age at treatment, age at first use, education and types of treatment settings (7). There is also further information available on drug use prevalence, overdose deaths, infectious diseases, and prices collected from governmental contact points (7). The GDS, as an independent research organization presents data in different areas related to drug-use patterns within segmented populations, alcohol consumption, risky drinking patterns, patterns of use, and harms of commonly used drugs (8). The GDS surveys are not expert-centered and gather data directly from drug-using populations. Leaning more toward treatment-centered surveys, in a joint collaboration, the WHO and UNODC have put together a basic suggested package for treatment planning and monitoring at a global level. This includes the International Standards for the Treatment of Drug Use Disorders, the Treatment Demand Indicator (TDI), the UNODC Treatment Quality Assurance Tool and the SUD Treatment Facility Survey. The SUD Treatment Facility Survey aims to map the treatment of SUDs in individual countries with data collected from governmental contact points (4). The results of this survey have been published in a joint publication with the EMCDDA reporting drug treatment systems in the Balkans (10). However, there are no global reports available from this survey yet. While clinicians, treatment programs and their organizations are among the first to respond to dynamic changes in the addiction crisis around the world, none of those above-mentioned studies collect data from these addiction experts at the global level.

The obstacle and the gaps mentioned above, demonstrate a need for building a trustworthy global expert network infrastructure to proceed with proven methodological strategies in information collection and expert elicitation from addiction clinicians to ensure input validation, data validation, quality, and integrity alongside developing global inclusion and diversity.

The International Society of Addiction Medicine (ISAM), an international fellowship of physicians with over 15,000 members from nearly a hundred countries, is uniquely situated to support fostering this expert elicitation infrastructure and addressing the informational gaps in knowledge regarding dynamic changes in addiction treatment demand and services globally. With forming the new ISAM product named as the ISAM Global Expert Network (ISAM-GEN) represented in this protocol paper, ISAM would be able to continue the path started back in early days of pandemic with running a successful global survey gathering experts’ opinions from around the world and potentially build on this endeavor to expand a network of diverse addiction experts globally in order to establish an international infrastructure for developing expert-centered studies.

### 1.2 Objectives

We intend to conduct international longitudinal survey studies using expert information and opinions as an important source of data to fill gaps mentioned above by collecting and verifying secure consistent data in various aspects of addiction medicine through different groups of participants. Thus, our objectives in running these surveys are:

Objective 1: To track substance use supply, demand, health impacts, co-occurring disorders/concerns and multiple morbidities, service provision, and policymaking by systematically recruiting and eliciting timely information from a global network of clinicians, treatment programs, and addiction societies. We intend to collect a comprehensive set of data with focus on potential first-line responders in addiction treatment services covering a detailed range of topics to track substance use and its trends longitudinally on a timely basis.

Objective 2: To measure future trends in new illicit substances, policies and treatment services from the lens of clinicians around the world. These data will prospectively help scholars and policymakers identify global substance-related challenges as they emerge. We intend to use cross-national surveys and expert elicitation tools to analyze and provide reliable and valid assessments of key indicators in addiction medicine. Our methodology will build on expert survey design and latent variable modeling techniques including highly adaptable models grounded in Bayesian item response theory.

Such studies will expectantly provide up-to-date and longitudinal global data about important trends in substance use and SUDs from the perspective of potential front-line clinicians and researchers. The data is intended to include opioid demand (prevalence estimates of substance use and SUDs, patterns of use, etc.) and supply (availability, accessibility, purity, price, amount, etc.), substance-related harms (health, social, legal and economic), policymaking priorities and attitudes, treatment services (mental and physical) for people with SUDs, dynamic trends and changes in substance use as perceived by addiction medicine professionals, forecasting knowledge on the future of SUD-relevant services/policies (e.g., harm reduction, decriminalization, or legalization) across the globe, and emerging crises including pandemics, new substances, rapid changes in opioid supply, disasters and forcibly displaced populations.

This project is being designed to expectantly lead to the establishment of a globally representative and validated system for detecting emerging changes in SUDs. Leveraging the knowledge of potential hundreds of clinicians and treatment programs as a front line of facing emerging changes will address important knowledge gaps in SUDs at the country-year level. This will be of particular importance in the case of emerging disruptions and crises around the globe that eventually influence drug addiction on a global level.

We also aim to provide estimated future trends and emerging crises with new substances or behaviors, treatment needs and services, drug policies, and national attitudes towards addiction treatment services based on expert opinions.

Here in this protocol paper, stemming from the necessity and rationales behind developing the ISAM-GEN, we sought to expand the methodological infrastructures and initial formation steps of the network in order to develop the cornerstone for the ISAM-GEN projects as a rigorous and transparent platform for the future steps ahead.

## 2 Materials and methods

### 2.1 Study design

Aiming for multi-phase, cross-sectional and longitudinal global survey studies, four main groups of (1) addiction societies/associations, (2) treatment programs, (3) individual clinicians and (4) related stakeholders (including but not limited to policymakers, program planners, members from the pharmaceutical industry, and networks or associations representing people who use drugs, etc.; with transparent management of all potential conflicts of interests after receiving Institutional Review Board (IRB) and ISAM Board of Directors approval) will be targeted as survey participants. Invited participants will represent a wide range of key informants in the field of addiction medicine. These groups will be contacted directly via the ISAM-GEN. Surveys will be presented through an online platform that allows participants to track their status on each running survey and previous surveys. The main aim is to develop and perform surveys on issues regarding SUDs and their treatment status across the world in a timely manner.

### 2.2 Survey development and validation

To ensure the content validity and significance of survey questions for this project, an adapted Delphi consensus technique has been devised to achieve expert consensus on gathering the most relevant questions in the field to be asked at national, international, and global levels. This technique relies on several rounds of commenting and/or item-rating rounds providing feedback to the participants. Building on Ekhtiari et al.’s approach, a steering committee (SC) will be in charge of developing the methodology and resolving conflicts in order to better handle the project flow by making final decisions (11, 12). The SC members will be recruited from the ISAM-GEN steering committee (https://isamweb.org/global-expert-network/). A larger group of individuals with expertise in addiction research will form an expert panel (EP) that will take part in the Delphi process along with the SC to include the opinions of a much broader and more diverse group of international experts in the process of survey development. This group will be recruited from a list of researchers most active in developing and conducting addiction, mental health, and public health surveys around the world based on the number of publications and authorship positions identified through performing a systematic review on the topic and searching the Expertscape database (https://expertscape.com).

Following the aims of the ISAM-GEN, survey content categories will cover different aspects of addiction medicine at local, national, regional, and global levels from the perspective of clinicians and their treatment programs and societies/associations. These will include drug demand, drug-related harms, policymaking priorities and attitudes including government and professional policies and their impact, health services for SUDs and behavioral addictions, trends and changes in addiction medicine, availability of related clinical guidelines, and rapid assessments for emerging crises. Multiple different techniques meant to enable robust expert elicitation will be utilized, including:

- The use of standardized vignettes to help individuals answer questions more reliably and also provide a standardized benchmark to allow for comparing of responses between experts.
- Questions to assess expertise in areas of interest for each survey, both explicitly (“how confident are you of your expertise?”) and implicitly (asking questions with known answers).
- The use of probability distribution visualizations on questions for which experts have to provide probabilistic answers.
- The use of item response theory to synthesize and interpret survey responses across experts.
- The use of qualitative consensus-building and opinion aggregation techniques, such as focus groups and Delphi-consensus procedures, where appropriate.
- Taking advantage of different incentivizing methods for promoting collaboration such as developing the scoring system in the survey platform.

Question types will vary based on the subject and what the study platform provides which includes multiple-choice, slider, text entry, matrix table, form field, rank order, net promoter score, graphic slider, pick and group and rank, drill down, constant summation, file upload, highlight, side by side, hot spot and heat map. Questions will be checked thoroughly to avoid any sensitive or confidential items being asked from the participants.

After developing a basic set of questions in the aforementioned categories by the SC, the initial draft of the questionnaire will be shared with both the SC and EP in order to perform a Yes/No rating for each question as to whether to consider the question in the final survey or not. Before the rating phase, participants are instructed to comment on modifying question types, question wordings, importing more questions in each category and adding any further recommendations. The SC will finalize the questionnaire based on the feedback received. Questions reaching a threshold of 70% consensus will be considered for inclusion in the final survey (11, 12).

### 2.3 Study/survey proposal submission

Members of ISAM-GEN or investigators outside the network will have the opportunity to submit their proposals with suggested surveys to use the ISAM-GEN platform. Submitted proposals will be initially reviewed within the SC of ISAM-GEN, externally reviewed and will be returned to the applicant in case of any revisions. The facilities and platforms within the ISAM-GEN will then be accessible to the principal investigator to submit the detailed protocol, survey, and IRB approval to run the survey.

### 2.4 Survey participants

#### 2.4.1 Societies/associations as participants

**Figure 1:**
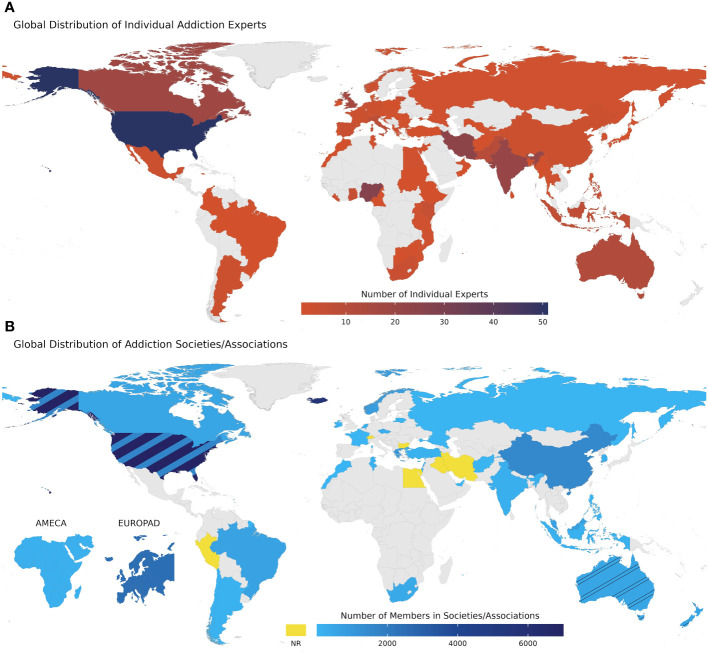
Global distribution of individual experts and addiction societies/associations within the network of ISAM-GEN. **(A)** Global distribution of individual addiction experts within the network of ISAM-GEN. This map represents the included experts up to the first wave of snowball sampling with the number of experts in each country. **(B)** Global distribution of addiction societies/associations within the network of ISAM-GEN. The number of members in the host country of each society/association is depicted in this map. Yellow shows countries with no available data on their number of members. Two smaller maps in the lower left corner of the figure show two societies/associations with regional scopes, including Africa & Middle East Congress on Addiction (AMECA) and European Opiate Addiction Treatment Association (EUROPAD). The remainder of the societies/organizations are abbreviated and named as follows: ASAM, American Society of Addiction Medicine; AAAP, American Academy of Addiction Psychiatry; CSAM, Canadian Society of Addiction Medicine; BAP, Barbados Association of Psychiatrists; ArSAM,Argentina Society of Addiction Medicine; ABEAD, Associação Brasileira de Estudos do Álcool e outras Drogas (The Brazilian Association for the Study of Alcohol and Other Drugs); SONEPSYN, Addiction Chapter of the Chilean Society of Psychiatry and Neurology; APAD, Peruvian Association of Addictionology; EUROPAD, European Opiate Addiction Treatment Association; FSWS, Maltese Foundation for Social Welfare Services, OKANA, Greek Organization Against Drugs; SAA, Icelandic Society of Alcoholism and other Addictions; VVGN, Vereniging voor Verslavingsgeneeskunde Nederland (Netherlands Society for Addiction Medicine); LPPA, Lithuanian Association of Addiction Psychiatry; FFA, Fédération Française d’Addictologie (The French Federation of Addictology); RPA, Russian Psychiatric Association; NAAC, Cyprus National Addictions Authority; CzMA, Czech Republic Society of Addiction Medicine; IRESAM, Irish Society of Addiction Medicine; NFRAM, Norwegian Society for Addiction Medicine; BMTA, The Bulgarian Methadone Treatment Association; ILSAM, Israeli Society of Addiction Medicine; AMA, Association Marocaine D’Addictologie (Moroccan Association of Addictology); STADD, Societe Tunisianne d’Addictology (Tunisian Society of Addictology); AMECA, Africa and Middle East Congress on Addiction; AEPA, Addiction Section, Egyptian Psychiatric Association; ISTA, Iranian Institute for Science and Technology of Addiction; ASAM, Afghanistan Society of Addiction Medicine; IRSAM, Iraqi Society for Addiction Medicine; LPS, Lebanese Psychiatric Society; NRC, National Rehabilitation Center; RANZCP, Royal Australian and New Zealand College of Psychiatrists: Addiction Faculty; AChAM, Australasian Chapter of Addiction Medicine; APSAD, Australasian Professional Society on Alcohol & other Drugs; APSI, Addiction Psychiatry Society of India; KAAP, Korean Academy of Addiction Psychiatry; AMAM, Addiction Medicine Association of Malaysia; PASS, Philippine Addiction Sciences Society; CADAPT, Chinese Association of Drug Abuse Prevention; IPA, Indonesian Psychiatrist Association; NAMS, National Addictions Management Service of Singapore. NR, Not Reported.

To ensure institutional representation, especially from underrepresented countries and regions, addiction medicine/psychiatric societies/organizations constitute the first group of survey targets. Members of these participants are accessible through the ISAM regional council (ISAM-RC) network via the regional representatives from Europe, Indian Subcontinent & South East Asia, Oceania, Canada, USA, Eastern Mediterranean (including the Middle East and North Africa), South America, the Caribbean and Central America, East Asia Pacific and Sub-Saharan Africa. As of February 1st 2022, ISAM had 27 affiliated societies/organizations worldwide and initiated a call for more societies/organizations to join the network after holding the first ISAM General Assembly of addiction medicine societies on February 10, 2022 (virtual), with participation of the 43 groups. Demographic data of all 43 societies (27 officially affiliated to ISAM and 16 non-ISAM affiliates) within the network of ISAM-GEN are represented in **Table 1**. The global distribution of these societies/associations/organizations is also depicted in **Figure 1**.

**Table 1:** Demographic information of societies/associations within the network of ISAM-GEN by February 2022.

No.	Addiction Medicine Society/Association	Abbreviation	Region	Organizational Structure	Affiliation	Establishment Year	Number of Members	Website
**1**	American Academy of Addiction Psychiatry	AAAP	North America	National	Affiliated	1986	1650	https://www.aaap.org/
**2**	American Society of Addiction Medicine	ASAM	North America	National	Affiliated	1954	7000	https://atest.asam.org/
**3**	Canadian Society of Addiction Medicine	CSAM - SMCA	North America	National	Affiliated	1989	500	https://csam-smca.org/
**4**	Argentina Society of Addiction Medicine	ArISAM	Central and South America	National	Affiliated	2020	67	http://www.arsamargentina.com/
**5**	The Brazilian Association for the Study of Alcohol and Other Drugs	ABEAD	Central and South America	National	Non-Affiliated	1970	600	http://www.abead.com.br
**6**	Addiction Chapter of the Chilean Society of Psychiatry and Neurology	SONEPSYN	Central and South America	National	Non-Affiliated	1932	74	www.sonepsyn.cl
**7**	Peruvian Association of Addictionology	APAD	Central and South America	National	Non-Affiliated	1996	NR	NR
**8**	Barbados & Caribbean Society of Addiction Medicine	BPA	Central and South America	National	Non-Affiliated	1994	30	NR
**9**	Moroccan Association of Addictology	AMA	Africa	National	Affiliated	2014	12	https://ama-maroc.com/
**10**	Tunisian Society of Addictology	STADD	Africa	National	Affiliated	2015	124	NA
**11**	South African National Council on Alcoholism and Drug Dependence	SANCA	Africa	National	Non-Affiliated	NR	NR	https://www.sancanational.info/
**12**	Africa and Middle East Congress on Addiction	AMECA	Africa	National	Non-Affiliated	NR	NR	www.iameca.org
**13**	Addiction Section, Egyptian Psychiatric Association	AEPA	Africa	National	Affiliated	1971	NR	http://www.epassociation.net/
**14**	Iranian Institute for Science and Technology of Addiction	ISTA	Middle East, Eastern Mediterranean & West Asia	National	Affiliated	2016	NA	www.http://istaweb.org/
**15**	Afghanistan Society of Addiction Medicine	ASAM	Middle East, Eastern Mediterranean & West Asia	National	Non-Affiliated	2022	20-30	http://www.epassociation.net/
**16**	Iraqi Society for Addiction Medicine	IRSAM	Middle East, Eastern Mediterranean & West Asia	National	Non-Affiliated	2007	NR	NR
**17**	Lebanese Psychiatric Society	LPS	Middle East, Eastern Mediterranean & West Asia	National	Non-Affiliated	1991	55 members (out of 75 active psychiatrists nationally)	NA
**18**	Addiction Psychiatry Foundation of Türkiye	BPV	Middle East, Eastern Mediterranean & West Asia	National	Affiliated	2002	110	NA
**19**	European Opiate Addiction Treatment Association	EUROPAD	Europe	29 European Countries plus Russia and Israel	Affiliated	1994	2500	https://www.europad.org/
**20**	Maltese Foundation for Social Welfare Services	FSWS	Europe	National	Affiliated	1994	NR	https://fsws.gov.mt/
**21**	Greek Organization Against Drugs	OKANA	Europe	National	Affiliated	1995	More 1000	www.okana.gr
**22**	Icelandic Society of Alcoholism and other Addictions	SAA	Europe	National	Affiliated	1977	5	https://saa.is/
**23**	Netherlands Society for Addiction Medicine	VVGN	Europe	National	Affiliated	1985	200	https://www.vvgn.nl/
**24**	Lithuanian Association of Addiction Psychiatry	LPPA	Europe	National	Affiliated	1998	20	www.lppa.lt
**25**	French Federation of Addictology	FFA	Europe	National	Affiliated	2000	20	http://www.sfalcoologie.asso.fr/; https://www.federationaddiction.fr/; http://www.addictologie.org/
**26**	Russian Society for Addiction Medicine	RPA	Europe	National	Non-Affiliated	2010	35	https://psychiatr.ru
**27**	Cyprus National Addictions Authority	NAAC	Europe	National	Affiliated	2001	100	https://www.naac.org.cy/el/home-en
**28**	Czech Republic Society of Addiction Medicine	CzMA	Europe	National	Non-Affiliated	1993	238	Umbrella Society: https://www.cls.cz/english-info Society on Addiction: https://snncls.cz
**29**	Irish Society of Addiction Medicine	IRE-SAM	Europe	National	Non-Affiliated	2022	10	NA
**30**	Norwegian Society of Addiction Medicine	NFRAM	Europe	National	Non-Affiliated	2005	800	https://www.legeforeningen.no/foreningsledd/fagmed/norsk-forening-for-rus–og-avhengighetsmedisin/
**31**	Addiction Psychiatry Society of India	APSI	South East Asia, Indian Subcontinent	National	Affiliated	2020	180	https://addictionpsychiatry.in/
**32**	Korean Academy of Addiction Psychiatry	KAAP	South East Asia, Indian Subcontinent	National	Affiliated	1996	200	https://addictionacademy.org/eng/
**33**	Addiction Medicine Association of Malaysia	AMAM	South East Asia, Indian Subcontinent	National	Affiliated	2006	700	http://www.amam.com.my/
**34**	Philippine Addiction Specialists Association	PASS	South East Asia, Indian Subcontinent	National	Affiliated	2022	10 core members	NA
**35**	Chinese Association of Drug Abuse Prevention	CADAPT	South East Asia, Indian Subcontinent	National	Non-Affiliated	NA	NA	cadapt.com.cn
**36**	Indonesian Psychiatrist Association	IPA	South East Asia, Indian Subcontinent	National	Affiliated	2016	100	PDSKJI.org
**37**	National Addictions Management Service of Singapore	NAMS	South East Asia, Indian Subcontinent	National	Affiliated	2008	87	https://www.nams.sg
**38**	Royal Australian and New Zealand College of Psychiatrists: Addiction Faculty	RANZCP	Oceania	Australia, New Zealand and Asia Pacific	Affiliated	NA	>300 psychiatrists and >120 trainee members	https://www.ranzcp.org/membership/faculties-sections-and-networks/addiction-psychiatry
**39**	RACP - Australasian Chapter of Addiction Medicine	AChAM	Oceania	National	Affiliated	2002	302 members (as of 2021)	https://www.racp.edu.au/about/college-structure/adult-medicine-division/australasian-chapter-of-addiction-medicine
**40**	Bulgarian Society of Addiction Medicine	BMTA	Europe	National	Non- Affiliated	2003	NR	NR
**41**	National Rehabilitation Center, UAE	NRC	Middle East, Eastern Mediterranean & West Asia	National	Affiliated	2002	31	https://nrc.gov.ae
**42**	Australasian Professional Society on Alcohol & other Drugs	APSAD	Oceania	Australia, New Zealand and Asia Pacific	Non- Affiliated	1981	500	http://www.apsad.org.au/www.apsdadconference.com.au
**43**	Israeli Society of Addiction Medicine	ILSAM	Europe	NR	Affiliated	NR	150	NR

NA, Not Available; NR, Not Reported.

More information on ISAM Regional Council membership and procedures is available in the ISAM-RC proposed terms of reference available on the ISAM website (https://isamweb.org/regional-council/).

#### 2.4.2 Collaborating treatment centers/programs as participants

As the second level of contact point for our surveys, collaborating treatment centers/programs will be considered one of the important sources of information for recruiting treatment-related data in the context of SUDs. In order to include as many centers/programs and survey participants as appropriate, different types of treatment facilities will be considered based on a prior known categorization including low-threshold services, specialized outpatient SUD treatment services, hospital-based residential SUD treatment services, non-hospital residential SUD treatment service, mental health care services, therapeutic communities and specialized social reintegration service (4). Each of these centers/programs with at least 10 currently active patients within their facilities or having a minimum of 50 admissions per year will be eligible to be included in the list of our survey participants. In order to gather reliable data, chief administrators will be contacted to participate in the survey as representatives of their centers/programs. Survey participants will provide details of their roles and clinical responsibilities and any potential conflicts of interest to be considered as relevant contextual information for data analytics and interpretation.

#### 2.4.3 Individual experts as participants

As the third group of survey participants, addiction medicine experts are targeted for the invitation to the ISAM-GEN. The inclusion/exclusion criteria for choosing the candidate experts follow the ISAM-GEN protocol, which is summarized as meeting at least one of the five following criteria below:

- Being a clinical practitioner who has provided services to over 50 patients with SUDs in the last 3 years prior to evaluation.
- Chairing, co-chairing, or vice-chairing an academic or research division/department/institution related to mental health, public health, or addiction in a country/state.
- Chairing, co-chairing, or vice-chairing a WHO collaborating center or WHO-affiliated office at the global or regional level.
- Having a vote/voice in decisions regarding policymaking for SUDs at the national/state level.
- Having relevant peer-reviewed publications in relevant areas.

**Figure 2:**
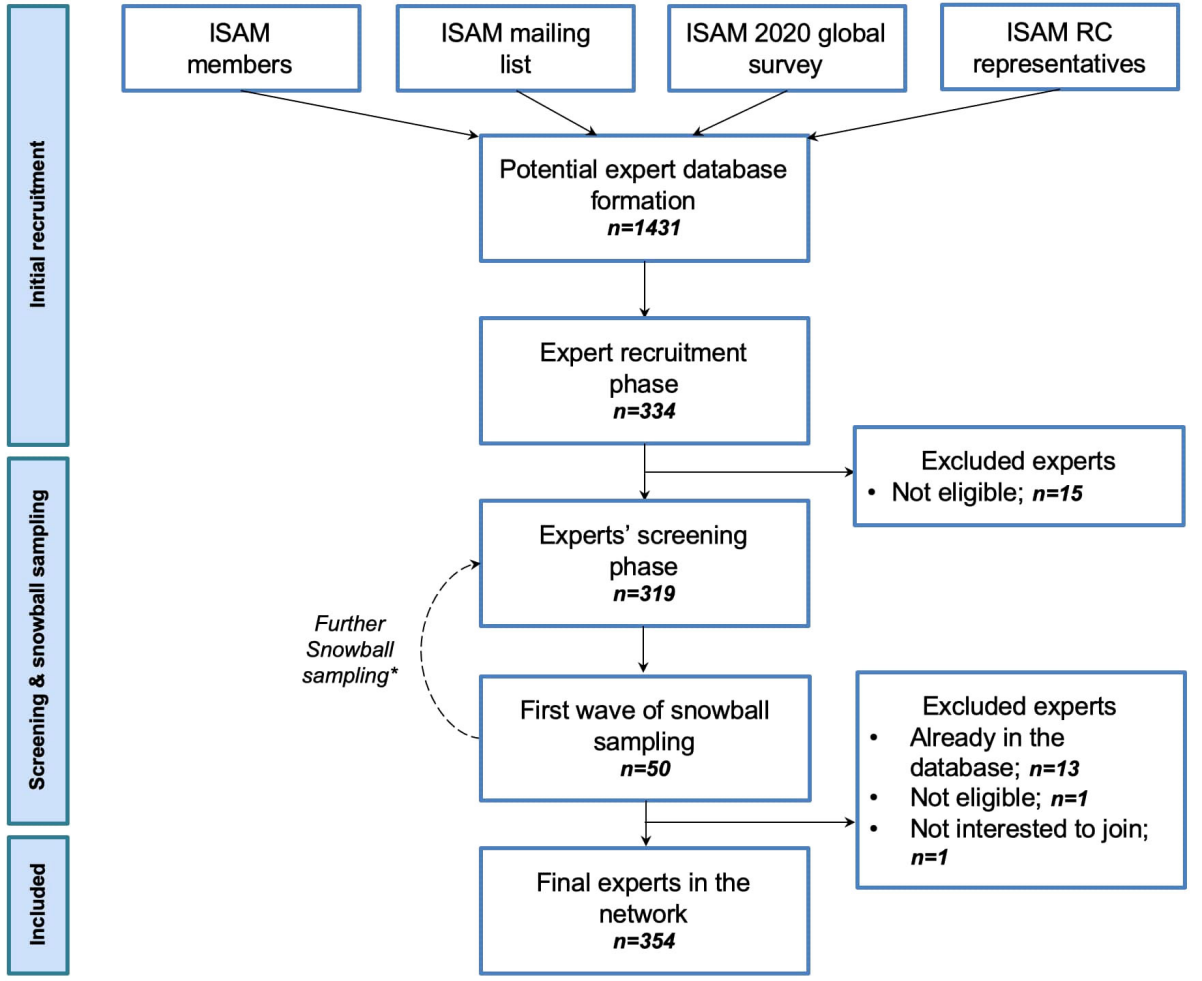
Expert recruitment process flowchart. The initial step of individual experts recruitment consisted of forming a mother dataset of contact persons (N= 1431), by approaching four pools of addiction medicine professionals, including ISAM members; ISAM mailing lists comprising annual ISAM conference, workshops, and examination registrants; addiction medicine experts who contributed to the ISAM 2020 global survey on COVID-19 and SUDs (2); and ISAM regional council representatives who were directly nominated by regional key informants affiliated with ISAM. Through the second step of the expert’s recruitment process, those who responded to ISAM-GEN’s first email (N = 334) were asked to consent to become a part of the network and submit more information about their experience and expertise in the field of addiction medicine for screening and evaluation purposes. Fifteen respondents were excluded at this stage due to their inability to fulfill the criteria (see section 2.4.3 for the criteria list). Eligible individuals (N = 319) were contacted again and were asked to nominate up to three addiction medicine experts from their own geographical regions and three from other regions. Through the first wave of snowball sampling of expert recruitment (September 2022), 50 experts were nominated. The nominated experts were then contacted iteratively (between the recruitment and screening phases). They were approached with the same survey questions for collecting their basic information for evaluation and screening purposes. After removing duplicates, screening for eligibility, and obtaining consent, 15 nominated experts were excluded from the final sample. As of the conclusion of the first wave of snowball sampling (January 2023), the final dataset of experts in the ISAM-GEN network consisted of 354 experts. The experts recruited via snowball sampling were again asked to nominate addiction medicine experts at national and international levels through the second wave of snowball sampling. ISAM: International Society of Addiction Medicine; RC: Regional Council. * The snowball sampling is an ongoing process until a saturation point is reached (minimum of 5 experts for each country/region). This saturation point will be defined based on the overlap in recommended experts and experts already considered for inclusion, but expert invitations may continue for countries not represented by at least ten experts in the network.

Recruitment of the experts will follow a multi-phasic process (**Figure 2**). The purpose of the process will be to formally include expert opinions in expert recruitment and to estimate the saturation of the pool of recruited experts based on expert endorsements of other experts using multiwave snowball sampling (13, 14). In the first phase of expert recruitment, an initial core of experts will be formed from the following groups of experts:

- Those nominated by the network of over 27 ISAM affiliate societies and other known regional representatives in 5 continents across the globe.
- Those among the participants in the 2020 global survey (Baldacchino et al., 2020) who indicate their interest in joining the ISAM-GEN and meet the inclusion criteria.
- ISAM members of WHO/UNODC collaborating centers, national research centers, universities, hospitals, ministries of health, think tanks, and non-governmental organizations (NGOs) who meet the inclusion criteria and are interested in joining the ISAM-GEN.
- ISAM members of the Arab League, African Union, EMCDDA, and other related addiction-focused intergovernmental institutions.
- Experts on the mailing lists of ISAM and several addiction science expert networks (1–3, 11, 12) who meet the inclusion criteria and are interested in joining the ISAM-GEN.

These initial seed experts will then be emailed and asked to recommend other experts who may meet the eligibility criteria from the countries they represent or others. This second wave of experts will again be appraised based on the inclusion criteria and those meeting the inclusion criteria will be invited to join the ISAM-GEN. This multi-wave snowball sampling will be repeated until a saturation point is reached (minimum of 5 experts for each country/region). This saturation point will be defined based on the overlap in recommended experts and experts already considered for inclusion, although invitations may continue for countries not represented by at least five experts in the network.

**Figure 3:**
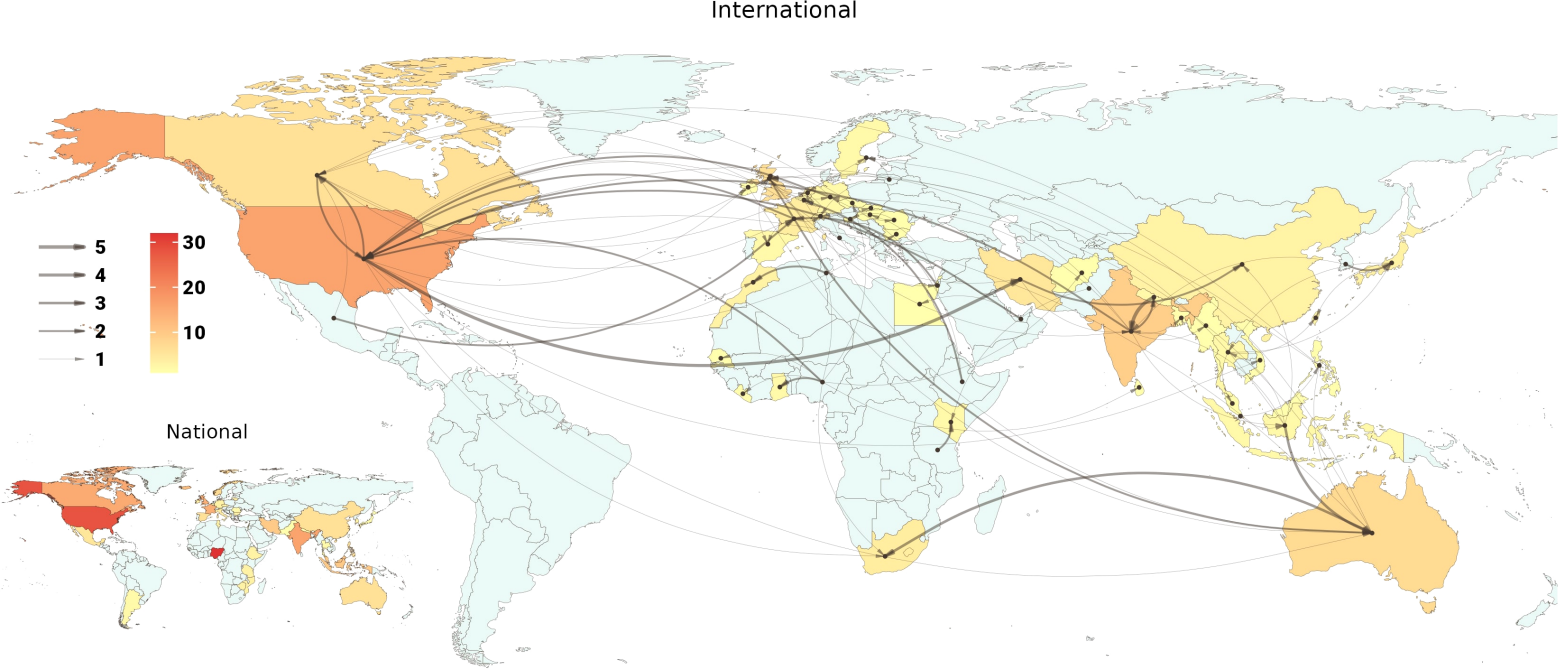
National and international snowballing network pattern on a global scale. This figure depicts the network of experts snowballing in two levels of international and national scale. In the international scale map, arrows start from the origin country of the nominator expert and end in the origin country of the nominated expert. The arrow width represents the number of nominations with a minimum of 1 and a maximum of 5. Country colors range from light to dark orange, representing the number of nominated experts from each country. The national map depicts the number of nominations within a single country, ranging from light to dark orange.

As a first step, a database of potential experts was formed based on the available list of experts mentioned before and following that, a first round of an expert recruitment survey was sent to a mailing list of 1431 individuals along with two reminders to non-respondents to the previous email. The third reminder was sent within 3 weeks after the first email. The expert recruitment survey was created on an online platform (Google Forms), consisting of 17 questions, primarily aiming to collect demographic, professional, and education-related data from the approached survey respondents. Respondents to the recruitment surveys entered the screening process conducted by a review committee (composed of AFJ, AKZ, FA and PR and supervised by HE) based on the inclusion criteria. Fifteen individuals were excluded after the screening because they did not fulfill the inclusion criteria. The remaining respondents were contacted with an additional survey that included 12 questions mainly related to potential scholarly publications of the experts, their affiliated addiction treatment center or clinic, and other addiction professionals they could potentially nominate to be recruited as experts in the ISAM-GEN. This survey initiated the first wave of the expert snowball sampling process within ISAM-GEN with a pool of 94 nominees nominated by 92 experts at a national level and 52 experts at an international level. From the initial pool of nominees, 50 experts responded to the invitation and recruitment survey accordingly. Out of them, 13 were already included in the database, one did not fulfill the criteria and one refused to be part of the network. This process has led to the inclusion of 354 experts in the network up to January 2023. The snowball sampling will be continued to reach the required threshold (minimum of 5 experts from each country). **Table 2** summarizes the demographic data of included experts. The global distribution of these experts is shown in **Figure 1**. **Figure 3** also shows the first wave of national and international snowballing network pattern on a global scale.

**Table 2:** Demographic and academic information for experts up to the first wave of snowball sampling (January 2023).

Demographic Variables	Experts (n=354) n (%)/Mean ± SD
**Gender** Male Female Prefer not to say Non-binary	223 (62.99%) 128 (36.16%) 2 (0.56%) 1 (0.28%)
**Continent** Asia Europe North America Africa Oceania and Australia South America	106 (29.94%) 81 (22.88%) 75 (21.19%) 71 (20.06%) 13 (3.67%) 8 (2.26%)
**Highest Academic Degree** Doctor of Philosophy (PhD) Doctor of Medicine and Philosophy (MD, PhD) Doctor of Medicine (MD) Master of Science (MSc) Bachelor of Science (BSc) Other	103 (29.1%) 97 (27.4%) 96 (27.12%) 38 (10.73%) 13 (3.67%) 7 (1.98%)
**Primary Discipline** Psychiatry Addiction Medicine Psychology/Counseling Drug/Health Policy Neuroscience Social Work Epidemiology Nursing Other Medical Specialties	118 (33.33%) 95 (26.84%) 65 (18.36%) 14 (3.95%) 10 (2.82%) 10 (2.82%) 9 (2.54%) 7 (1.98%) 21 (5.93%)
**Primary Affiliation** University/Teaching Hospital or Institution Government Health Program Independent Clinical Institute/Center Private Practice Independent Hospital Independent Research Institute	237 (66.95%) 52 (14.69%) 20 (5.65%) 17 (4.8%) 14 (3.95%) 14 (3.95%)
**Main Field of Activity** Providing Direct Clinical Practice/Support Researcher/Scientist Administrator/Executive/Manager Policy Maker	162 (45.76%) 148 (41.81%) 29 (8.19%) 15 (4.24%)
**Time Spent in the Field of Addiction (Years)**	16.23 ± 10.13
**Number of SUD Patients within the Last 3 Years of Practice (For Clinician Experts only, n=218)**	607.12 ± 933.98

To incentivize expert membership in the ISAM-GEN, expert members will be identified formally on the ISAM website. These experts will be offered ISAM membership (if eligible) fee waivers for a year after joining the ISAM-GEN. Members can also contribute to ISAM-GEN publications as authors.

#### 2.4.4 Related stakeholders

This group of experts (including but not limited to policymakers or program planners, individuals from the pharmaceutical industry and those from networks or associations representing people who use drugs, etc.) will be invited to the specific parts of the global surveys that are relevant to their field of expertise. The inclusion and exclusion criteria will be defined based on the specific requirements of each survey.

### 2.5 Study platform

Online integrated surveying system of ISAM-GEN is a developing platform which aims to provide participants (experts, societies/associations, treatment facilities) an infrastructure for running projects and surveys. The platform is intended to build upon a cloud-based software as a service (SaaS) architecture. The system will provide membership system along with online survey management and expert elicitation platform which also supports data analytics and provides online and live data visualization allowing users to compare survey results of their own with other countries and societies along with the ability to connect with other experts to address discrepancies and issues. We are also intending to design a scoring system for all levels of participants in order to promote collaboration and participation within the defined projects and also incentivize the process of rigorous and transparent data sharing and networking. Further details on data security and confidentiality are provided in the following sections.

### 2.6 Data collection procedure

Based on the survey subject and the chosen target group – experts, treatment programs, societies/associations, or related stakeholders - informants will be invited to participate in the survey by email invitations sent via the official email address of the ISAM-GEN. After accepting the invitations, more instructions on purpose, structure, and guidelines for completing the survey will be delivered. All participants will be informed of the voluntary nature of this research project by signing the consent form before joining the study. Participants will be invited to join the authorship list of the derived articles describing empirical data results as appropriate. The responses will be recorded in a private internet database which will be only accessible to the core survey research team. The surveys will consider various strategies to increase adherence including instructional videos, discussion webinars, regular reminders, translating questionnaires to other languages, etc.

### 2.7 Statistical analysis

Statistical analyses will be conducted according to a multistep plan. The data analyses plan will include the following:

- Data cleaning of the online dataset with quality control.
- Descriptive statistics of the general characteristics of the recruited sample, in terms of country of residence, discipline, age, gender, and educational level.
- Calculation of responses’ agreement for each country when there are two or more responses.

Descriptive data will be presented as Mean ± SD for each country’s response and the average of regional and global responses. Categorical variables for data related to each country will be presented as percentages. All statistical analyses will be performed using RStudio. Intraclass correlation coefficient 3k (ICC3k) analysis will be conducted with the aid of the ‘psych’ package (Northwestern University, Evanston, Illinois, USA, http://CRAN.R-project.org/package=psych v. 1.6.4.) to calculate agreement, if any, between multiple responses recorded from each country or each category of respondents, separately.

We intend to use tools for expert elicitation and cross-national survey design and analysis—developed in ecology and political science expert elicitation studies but new in addiction research to our knowledge—to provide valid and reliable assessments of key indicators in addiction medicine (15, 16). Protocols of running such studies within these areas have shown the possibility of providing enough incentives for the participants to participate in the surveys. The Varieties of Democracy project uses local experts and national coders to code a handful number of cases and asks raters to code neighbors/hubs, and then requests experts to complete anchoring vignettes (15). In the following, they apply item response theory models to leverage different types of knowledge, systematically aggregate ratings within cases, anchor observations and raters to the same scale, estimate/adjust for rater reliability and confidence in ratings. Our methodology will build upon expert survey design and latent variable modeling techniques—including highly adaptable models grounded in Bayesian item response theory—developed for the Varieties of Democracy project (15), which provides global measures of democratic institutions now routinely used both by scholars and policymakers, including the European Commission, World Bank, and USAID.

### 2.8 Publication and dissemination strategy

The results of these surveys will be disseminated by (a) peer-reviewed publications, (b) ISAM annual congresses as part of the reports from the societies in the ISAM-RC general assembly and (c) discussions with policymakers and international organizations. In this way, experts and societies/associations will be encouraged to respond consistently and accurately to the surveys.

### 2.9 Pilot study

A pilot study targeting opioid use disorders with categories on the prevalence of opioid use, opioid use treatments, opioid use harm reduction, opioid use legalization and opioid use stigmatization will be performed involving addiction societies/associations via the connections through the ISAM-RC. This study will also report a snapshot of the addiction societies/associations worldwide and facilitate streamlining the executive process. ISAM-GEN officers and SC will design the questions of the pilot study.

### 2.10 Ethics and funding

#### 2.10.1 Ethics approval

The protocol draft has been approved by the Research Ethics Committees of the National Institute for Health Research, Tehran University of Medical Sciences (IR.TUMS.NIHR.REC.1401.007).

#### 2.10.2 Data confidentiality

The obtained data will be fully identifiable only to the investigators of this study and selected ISAM-GEN staff. This will enable personalized revisions to be made to the questionnaire. The participants’ personal information or observations will not be used without informed consent. In cases of involvement of researchers other than the investigators of the study during data analyses, they will be provided with fully anonymous data only. Participants will be asked via email for their consent to be quoted in this study’s publication and in subsequent publications. Publications will provide results in a totally de-identified summary form, making it impossible to identify any individual responses.

#### 2.10.3 Storage of data

The data will initially be stored on servers maintained by the preferred online platform. After completing the online questionnaire, responses will be downloaded and stored on secure local servers at the hosting universities/academic institutions. Once done, the data will be removed from the online platform. Data will be stored at the survey’s hosting university/academic institution for a minimum of seven years. Only the investigators of this study will have access to the data. The investigators will retain the data if there is a continued analysis of the dataset after a seven-year period. Otherwise, the data will be deleted.

#### 2.10.4 Use of data for other purposes

All participants will be provided access to a de-identified group dataset summarizing the results of the study. The investigators may also use the data as part of their research. When the research results are published in a thesis, or journal, or presented at scientific conferences, no information will be included that reveals participants’ identities without their prior permission. Only aggregate de-identified data may be used for other projects after approval for ethics.

#### 2.10.5 Possible benefits and risks to participants

Participants will receive no direct benefits from taking part in this research. It is expected that participation in this study will pose no threat to participants’ well-being. As experts in the field, the items on the questionnaire are highly likely to be items with which participants are familiar and that they encounter in their daily work. Nevertheless, participants are encouraged to withdraw participation if participation in this study causes distress or discomfort.

#### 2.10.6 Potential conflicts of interest

All the investigators involved in designing the surveys, analyzing the data, interpreting the results and publishing the reports will need to report any potential conflict of interest in advance and inform the principal investigators if there is any update in their conflict of interest within 3 months.

## 3 Discussion

The ISAM-GEN initiative attempts to address several systematic information gaps around dynamic changes in supply, harm, and treatment services related to different substances worldwide. Compared to existing platforms for implementing global projects and surveys in the field including WHO, UNODC, EMCDDA and GDS which mostly rely on governmental reports, ISAM-GEN surveys and studies will depend on expert opinion on the three levels of societies, associations/organizations, collaborating treatment centers/programs, and individual experts through expert elicitation methods, specifically in areas where data in the field is scarce and there is a need for time-sensitive, proactive actions to be taken under consideration (4–8). Meanwhile, future trends in the field may also be inferred based on experts’ perspectives to be considered at national, regional and global policy-making levels.

The initial steps in developing the network have culminated in the collation of a potential expert database of 354 individuals from 78 countries worldwide. These experts have participated in a snowball sampling process which has allowed us to estimate the network connectivity between their respective countries, in **Figure 3**. This has shown that there are countries where experts are more advanced in networking with foreign points of contact such as the USA and European countries while the pattern appears less enriched within the African countries. Forty-three addiction societies/associations also accepted to join our network and contribute as standalone reference points in our studies. Collaborating treatment centers/programs will be recruited as another source for data collection stemming from our previously mentioned (section 2.4.2) points of reference in upcoming steps. Altogether, building these branches of expert reference points will bring together an extensive collaborative network of addiction experts on a global scale, allowing the field to stay connected on different levels and synthesize valuable expert opinions in a structured manner.

Leveraging the knowledge of hundreds of different types of experts at the frontlines in facing emerging changes will address important knowledge gaps in SUDs at an international level. Meanwhile, we foresee some obstacles in developing the ISAM-GEN. Although the network allows for building upon a fundamental global platform for addiction experts to share first-hand experiences, some regions need to be more represented and we need full global representation within the network. We believe expert snowballing methods will allow for a more diverse group of experts to join the network in the near future through the next rounds of snowball sampling. Another inherent limitation is that expert judgments may not always be the best points of reference, and are second at the bottom of the evidence pyramid (17). However, in cases of data scarcity and time-sensitive challenges in the field, it provides an important resource. As defined precisely in the methods section, we intend to take advantage of expert elicitation methods to design cross-national surveys within the network in order to provide information on such critical circumstances when needed.

This project will provide a globally representative and extensively validated system for detecting emerging changes in SUDs. We believe the ISAM-GEN initiative will form an efficacious infrastructure for addiction medicine experts around the globe to mitigate the burden of challenges the field faces across different cultures and borders. The ISAM-GEN will facilitate data collection by taking part within a network designed for conducting surveys and studies or submitting proposals for providing global perspectives on specific topics.

## Ethics statement

The protocol draft has been approved by the Research Ethics Committees of the National Institute for Health Research, Tehran University of Medical Sciences (IR.TUMS.NIHR.REC.1401.007). All participants will be informed of the voluntary nature of this research project by signing the consent form before joining the study.

## Author contributions

HE, MP, AMB, AS, and AZ contributed to the conception and design of the protocol. AZ, PR, and FA contributed to data collection and organized the databases. ME and AZ analyzed the data, ran the statistical analyses, and designed the figures. HE, MP, AMB, and DPe supervised the analysis and gave conceptual advice. AZ, PR, AS, and HE contributed to drafting the first draft of the manuscript. MP and AMB edited the manuscript. All authors contributed to the manuscript revision, read, and approved the submitted version.

## References

[1] Lemahieu J-L Me A. World Drug Report 2021. United Nations publication, Sales No. E.21.XI.8. Vienna, Austria: UNODC (2021). Available at: https://www.unodc.org/res/wdr2021/field/WDR21_Booklet_2.pdf.

[2] Farhoudian A Radfar SR Mohaddes Ardabili H Rafei P Ebrahimi M Khojasteh Zonoozi A . A global survey on changes in the supply, price, and use of illicit drugs and alcohol, and related complications during the 2020 COVID-19 pandemic. Front Psychiatry. (2021) 1134. doi: 10.3389/fpsyt.2021.646206 PMC8377291 34421664

[3] Radfar SR De Jong CA Farhoudian A Ebrahimi M Rafei P Vahidi M . Reorganization of substance use treatment and harm reduction services during the COVID-19 pandemic: a global survey. Front Psychiatry. (2021) 12:349. doi: 10.3389/fpsyt.2021.639393 PMC8135096 34025471

[4] WHO/UNODC Substance use disorder treatment facility survey (2018). Available online at: https://www.unodc.org/documents/WHO_UNODC_Facility_survey_Draft_for_field_testing_March_2018.pdf.

[5] UNODC Documents . World Drug Report 2021 Methodological Annex. Vienna: Research and Trend Analysis Branch, UNODC (2021).

[6] UNODC Documents . Global Assessment Programme on Drug Abuse (GAP), Annual Reports Questionnaire (ARQ). Vienna: UNODC. (2000).

[7] Statistical Bulletin . Datasets. Lisbon, Portugal: EMCDDA (2021). Available at: https://www.emcdda.europa.eu/data/stats2021_en.

[8] Winstock A Barratt M Ferris J Maier L . Global drug survey. London, United Kingdom: GDS Core Research Team (2017). Available at: https://www.globaldrugsurvey.com/.

[9] Substance Abuse and Mental Health Services Administration, National Survey of Substance Abuse Treatment Services (N-SSATS): 2020 . Data on Substance Abuse Treatment Facilities. Rockville, MD: Substance Abuse and Mental Health Services Administration (2021).

[10] Drug treatment systems in the Western Balkans: Outcomes of a joint EMCDDA-UNODC survey of drug treatment facilities. EMCDDA, UNODC. Available at: https://www.unodc.org/documents/drug-prevention-and-treatment/EMCDDA_UNODC_Publication.pdf.

[11] Ekhtiari H Zare-Bidoky M Sangchooli A Janes AC Kaufman MJ Oliver JA . A methodological checklist for fMRI drug cue reactivity studies: development and expert consensus. Nat Protoc. (2022) 4:1–31. doi: 10.1038/s41596-021-00649-4 PMC9063851 35121856

[12] Ekhtiari H Ghobadi-Azbari P Thielscher A Antal A Li LM Shereen AD . A checklist for assessing the methodological quality of concurrent tES-fMRI studies (ContES checklist): a consensus study and statement. Nat Protoc. (2022) 4:1–24. doi: 10.1038/s41596-021-00664-5 PMC7612687 35121855

[13] Christopoulos D . Peer esteem snowballing: a methodology for expert surveys. In: Proceedings of the Eurostat Conference for New Techniques and Technologies for Statistics. Bristol: University of the West of England (2009). pp. 171–9.

[14] Frank O . Survey sampling in networks. In: The Sage handbook of social network analysis (New York, USA: SAGE Publications Ltd), vol. 18. (2011). p. 389–403.

[15] Coppedge M Gerring J Knutsen CH Lindberg SI Teorell J Marquardt KL . V-Dem Methodology v11. V-Dem Working Paper forthcoming. (Gothenburg, Sweden: University of Gothenburg, V-Dem Institute) (2021).

[16] Choy SL O'Leary R Mengersen K . Elicitation by design in ecology: using expert opinion to inform priors for Bayesian statistical models. Ecol. (2009) 90:265–77. doi: 10.1890/07-1886.1 19294931

[17] Shaneyfelt T . Pyramids are guides not rules: the evolution of the evidence pyramid. BMJ Evidence-Based Med. (2016) 21:121–2. doi: 10.1136/ebmed-2016-110498 27405600

